# A National and International Medical Degree

**Published:** 1886-05

**Authors:** R. J. Nunn

**Affiliations:** President, Savannah, Ga.


VOL. III. MAY, 1886. No. 3.

### Original Communications.

### A NATIONAL AND INTERNATIONAL MEDICAL DEGREE.

AN INAUGURAL ADDRESS DELIVERED BEFORE THE MEDICAL ASSOCIATION OF GEORGIA, AT AUGUSTA, 21ST APRIL, 1886.

BY R. J. NUNN, M. D., PRESIDENT, SAVANNAH, GA.

It is self-evident that in this world the most precious possession of a human being is life. By Him who spake as "never man spake," the yielding up of life for an object was considered the greatest sacrifice which the most fervent love could suggest. "Greater love hath no man than this, that a man lay down his life for his friends;" but the prolongation of life, and certainly its pleasures, are dependent upon a condition of health, and hence to the individual the value of health is second only to that of life itself; indeed, few of us there are who have not had positive evidence that in many instances life without health becomes an intolerable burden.

As communities are but the multiplication of the individual, the conclusion is unavoidable that the prosperity, nay, the very existence of a nation is dependent upon the life and health of its people.

It follows, then, that the highest duty of a government is to protect and care for the life and health of those it controls.

Far more important than tariffs or currency is the life of the people. No questions of party ascendency can compare in importance with those affecting the health of the masses. Hygiene and sanitation must ever occupy the first place in the minds of wise, prudent and far-seeing legislators. Add but one day to the life of each individual of the population of the United States and the gain to the country at large would be 136,986 years—equal to the lives of 3,913 men averaging 35 years each. Assuming the average value of a day's labor to be $1.00, this would amount to $50,000,000 as the actual money value of one day lost to the productive industry of the country; add now to this the cost of the care of the sick at a hospital rate of $1.00, and we have $100,000,000 as the actual money value of a day of national health; but when these days come to be multiplied into years the sum becomes too vast to be within the grasp of the comprehension of an ordinary mind, yet this boon is within the reach of a nation which pays proper attention to hygiene.

It is probable that in this country human labor is more valuable than in any other, and hence here, if anywhere, if we had wise and provident rulers, would this branch, at least, of medical education be pushed to its highest level.

It is evident that the class of men who devote special attention to the means of preventing and curing disease, and of prolonging the term of human life, becomes a most important factor in the national existence, and the proper education of the individual, for the profession they have adopted would receive most careful consideration, and would be an object of constant solicitude on the part of a wise and conscientious government. It speaks not well for legislatures when these matters have to be forced upon their attention by the physicians themselves.

Older by far than our language is the proverb, "*salus pupuli suprema est lex*," but our legislators seem to have forgotten it in this our day.

Granting these propositions to be true, we would expect to find older, wiser, better educated and more thoughtful communities dissatisfied with the amount of technical education which is quite satisfactory to younger, less experienced, less cultivated and less thoughtful nations. Among the former we would naturally look for laws tending to prevent the influx of physicians whose educational acquirements do not come up to the standard the government requires; and so we find in different nations laws regulating the practice of medicine graded in stringency according to the ideas of the rulers. Looking at home, for instance, we see that twenty-nine States have passed laws of this character, in five of which diplomas are not recognized as evidence of competency, and all persons, whether graduates or not, are subject to an examination. These five are Alabama, Arkansas, Mississippi, North Carolina and Virginia. In the other twenty-four States diplomas are recognized as an evidence of fitness to practice medicine. These are Arizona, California, Colorado, Delaware, Florida, Illinois, Kentucky, Louisiana, Michigan, Minnesota, Missouri, Nebraska, Nevada, New Hampshire, New Jersey, New Mexico, New York, Ohio, Pennsylvania, South Carolina, Texas, Vermont, West Virginia and Wyoming. Georgia has been forgotten in the above list, and for her we grieve to acknowledge that from having once had at least the semblance of an examining and licensing board, and ranking with the advanced five, she has, within the last few years, by the act of her legislature, taken place in the ranks of the four-and-twenty black-bird supporters of the "free for all" race in medicine.

The issuing of diplomas by medical examining boards, and the requiring of them by governments as guarantees of medical education, are evidences that the public is not capable of judging of the competency of physicians to practice their profession.

It is unfortunately too true that in every country in the world, perhaps without exception, there exists one or more colleges or licensing bodies ready to grant diplomas to persons notoriously incompetent, rather than lose the fee accruing to them for the diplomas, or else, owing to the peculiarities of their organization, the questions asked are so much a matter of routine that they become well known to parties who make a business of preparing candidates for licenses, and as a consequence the persons passing the most successful examinations are not unfrequently wholly incompetent to practice their profession.

It is a fact deeply to be regretted that business success in the medical profession most frequently attends those least educated; fortuitous circumstances, family connections, social surroundings, political influences and other favoring fatalities often bring extensive and yet unmerited practices, but when to these is added the usual capital of the charlatan, unblushing audacity, endless pomposity, self-assumacy and vanity, with bland and insinuating manners, success is almost certain.

Two things, then, should be demanded by the public, or by the government for them: 1st. That physicians should be thoroughly educated up to the highest standard attainable at the time of examination. 2nd. That the mode of examination and the constitution of the examining board shall be such that a diploma issuing from it will be absolutely positive evidence of the ability of holder to practice medicine in accordance with the latest teachings of the profession.

Medicine may be said to occupy a position on the border-land between the sciences and the arts.

It is evident that certain results can never be deduced from factors constantly changing without rule. In medicine there exists not a single element which is subject to any known law, which is certain or controllable; patients differ in all the essentials of life; idiosyncrasies are co-numerous with the individuals; diseases, although known by the same name, never run exactly the same course; the domestic relations are different for each patient; the hygienic surroundings are not identical; the climate varies; nothing can be more uncertain than the psychological influences surrounding individuals and exerting upon them powerful influences for evil or for good; the food is different; medicines vary in strength and in therapeutic effect, and so with the action of every element which comes within the province of the physician; hence there can be no certainty in medicine; no result can be foretold; nothing but careful watching and the ability to detect slight changes, and to meet them, will lead to success.

As a consequence the claim that medicine is an exact science is utterly untenable. There is no sure deduction to be made from any one symptom; there is no certain result to be foretold for any remedy. The more accurate the observer and the wider his therapeutic experience the better the physician. Theory in medicine is the parent of quackery; the quack has always ready a plausible reason for everything that happens and for everything he does; it requires a large experience to elevate a physician to the plane where he can as a reply to a question say, "I do not know." It requires years of disappointments, theories upon theories, acquired and rejected, to arrive at the inevitable conclusion that medicine is organized empiricism, the experience of the past arranged and tabulated for our guidance in the future, that as a rule the closer we keep to facts and the further we keep from theory the better physicians we are.*

From this rapid review of the factors with which a physician has to deal, it will be evident that his education should be of a special character, consisting not alone of such reading and technical knowledge as is usually required, but in addition a system of training in the use and application of his faculties to that rapid, accurate and minute observation which can only be acquired by years of tutelage under experienced observers.

The physician must be trained to accept as a guide or suggestion whatever appears reasonable, but to believe nothing until he has applied to it the test of practice; he must formulate no creed upon the experience of others, but must be unto himself a law; he must distrust books and believe only what he sees.

It has become a habit—indeed, it is quite the mode now—to undervalue the medical educational requirements of American colleges, to decry the educational facilities of our home institutions and exalt those of Europe. Never was there a greater mistake. The best place to study medicine is where the physician will practice, among the types of disease he will be called upon to treat.

---

*See Code of Ethics of Am. Med. Asso., On duties of Physicians in regard to consultations—paragraph 1.

For the man of brains the educational facilities of America are quite equal to any in the world, and are better suited to persons whose professional life is to be spent here. Original thinkers are just as numerous in America as elsewhere; vigor of thought is more usual here than on the other side of the Atlantic. Originality of conception is to the manor born. Brilliancy and dash in execution are the birthright of America.

Here we are not trammeled by the effete traditions of a by-gone age. We think and act independently.

Coolness and self-reliance are among the most valuable qualities of a practitioner, and should be assiduously cultivated. They are peculiarly characteristic of the American physician, and are the natural outcome of the isolated position occupied by such a large number of country practitioners, who in emergencies are required to render all sorts of professional service without the possibility of a consultation, and often without ordinary appliances being accessible; hence one of the best positions for a young man who has obtained his "license to study" is that of a "country doctor." A few years spent in country practice will teach him to respect his hard-working country brother, and will also teach him to depend upon his own resources, will make him wonderfully independent of professional aid, and will educate him up to the point where he can meet grave responsibilities alone and unaided.

These are some of the characteristics of our people, and these can best be cultivated at home.

Few of our young men are experts in foreign languages, so that much of the time spent on the continent is taken up in acquiring the language of the country; it follows that during that time little or nothing can be done at medicine. For social reasons, as a good send-off in business, it is all very well, but for a medical education I believe that for Americans as good can be had in America as in any country in the world.

The home of McDowell, and Sims, and Mott, and Gross, and Flint, and of our own Long, and a host of others equally honorable, is certainly no bad school; the birthland of anesthesia, of ovariotomy, and we may say of gynecology, cannot be wanting in originality.

In the case of a physician of some years' practice, where there is experience enough to appreciate the individualities, the peculiarities of each school, when the mind is capable of making useful comparisons of the various methods met with, then indeed a few months or years passed in such observations might and would be exceedingly useful.

If we look around us and carefully examine the conditions of various communities, we cannot fail to be struck with the fact that where the people are the most ignorant, where human life is of the least value, there will medical education be at a low ebb; and as a country rises in the social scale, or if those controlling the legislation of the country advance in learning and refinement, so will a high order of medical education come to be required. This is especially remarkable on the continent of Europe, among those governments whose national existence depends upon the number of their soldiers, and where of necessity the life and health of the man is of great importance to the community.

Would we see how a scientific, a high order of education is appreciated by our people, look at the millions spent by the governments of Europe to support universities, and compare this expenditure with the few dollars grudgingly appropriated here. See how scientific investigation is stimulated, fostered and rewarded by the rulers there, and how they are neglected here. There learned men are honored, and position and emoluments are open to them; here the scholar may beg his bread, while the lowest grade of a petty pot-house politician is petted and pampered and paid.

When scholarship and learning are esteemed so little, we need not wonder that we are no better than we are, but rather that we are as good as we are.

It is quite true that formerly the claims of physicians to an equal share of the honors conferred upon other learned professions were not tangibly acknowledged in some European countries, but of late years a change has taken place in this particular, and we find the medical profession gradually attaining to a position where its just claims will be acknowledged.

England has had to contend with a state of things very similar to that which at present exists in America; but there some years ago a *general medical council* was formed to take charge of the matter of examination, and an official directory is annually published of persons authorized to practice. It must not be imagined that the course of this council has been smooth, or that the standard of education is fixed. On the contrary, there has been much opposition to its plans, and from unexpected sources, viz., from the colleges themselves. The interests of some of them were jeopardized and their authority would be curtailed, their freedom of action limited, and they opposed the change. Much, it is true, has been done, but much more remains to be accomplished.

America, with a civilization of a couple of hundred years, has been often taunted by Europeans with practically selling medical diplomas; but let me tell you and remind our accusers that in their own communities, under their own governments, hoary with the experience of a thousand years, a half century has not elapsed since diplomas were purchasable from colleges and universities of standing—nay, if I recollect correctly, were publicly advertised by agents. It is little more than a quarter of a century since fellowships of royal colleges were offered for sale to members of a certain number of years of membership; and even within the last decade dental diplomas were offered for sale by circular. Indeed, a few years ago, and perhaps at this very moment there may be practicing in England men whose sole qualification is that of being ancient practitioners.

When we take into consideration the disparity in years, we have no reason to shrink from a comparison, but we may rather be proud of the position we have attained in medical education.

In a business point of view, the mere possession of a better medical education will not be likely to insure to the possessor a success superior to that accorded to the veriest professional mountebank, unless there is at hand some mode of presenting the fact to the public; and to attain this end no other means suggests itself than the establishment of a degree, the requirements of which shall equal the highest demands of modern civilization, and surrounded by such safeguards as to be unattainable by the uneducated, even though backed by money, by social position and political influence.

Seemingly these results are attainable through two channels only: 1st. Professional influence directly and indirectly exercised; 2d. Legislative enactments.

The medical educational institutions and the licensing powers of this country are virtually in the hands of the profession, but, alas! medical sectarianism has so divided our ranks that little good can be expected from this source; still it is not impossible that a degree could be established by agreement between associations and a system of representation by which, upon receiving a diploma, the holder would acquire the right to practice in the States represented. Such a system, however, would be neither as simple, as economical nor as authoritative as a national and an international medical degree.

Experience has shown that there is little prospect of obtaining in the near future from State legislatures any general legislation regulating medical education which will be of any practical value.

While much has been done, and more probably will be done in some States, there are others in which there is little hope that any advance will be made for years to come, and even if the laws required were enacted they would be, through the apathy of officials, of no avail in many States, as they are practically ignored in some where they already exist, and where it has been found difficult, if not impossible, to obtain a verdict under the existing laws, or a verdict being obtained to enforce the penalty.

The legislators represent the people, and interested parties, actuated by selfish motives, have led the more ignorant, and hence the more easily deceived classes into the error of believing that this would be class legislation for the benefit of the doctors and to the detriment of the people.

Great conflicting interests will be opposed to the regulation of medical education by State laws: "the homœopathists, the physio-medical, the hygeio-therapeutic and the mixed schools included in the enumeration of the legally acknowledged institutions in the United States in the Report on Medical Education of the Illinois State Board of Health for 1885," will resist it; then the eclectics will oppose it. "The Eclectic Medical Society of the State of New York has declared formally against all attempts at placing legal restrictions on the practice of medicine, and the President of the Eclectic Medical Association of Pennsylvania declared in his annual address that the principles of eclecticism are based mainly on the 'American principle of freedom.' . . . Their creed is, let any one practice who wishes to do so; let him get his diploma as he wishes, and let him help his practice with as much advertising as he desires." Med. Rec., Nov. 14, 1885, p. 546–7.

With such principles it is evident that the eclectics can be largely recruited from the illiterate classes, and will exert among them a great influence, and as these constitute the great bulk of the voters to whom the successful politician must cater, it follows that bills regulating technical medical education must be a dead letter until this eclecticism becomes a thing of the past.

The homœopaths of the N. Y. County Homœopathic Medical Society have also reported against the bills for the appointment of a State Board of Examiners for the purpose of licensing physicians. Med. Rec., Feb. 20, 1886, p. 215.

"The peculiarities of our political system are also a serious difficulty to be encountered. The morals of party organization are too well known to be trusted for a moment, in my estimation, to make a wise selection of health officers for the public or to guard the entrance to the practice of medicine. . . . The boards are painfully incompetent, and as the position is a political one can never be better under the present form of government."—Coll. & Clin. Rec., Vol. vi, No. 12, p. 232.

As an improvement upon the present system, or rather want of system, of granting medical licenses, it is probable that the great majority of thoughtful physicians will favor examining boards, the members of which shall be unconnected with educational institutions; it would be wise, however, to act cautiously in the matter of unreservedly endorsing the establishment of an independent State Examining Board in each State to examine the ultimate results which would come from such a system, and determine whether a better system cannot be proposed to correct the evils undeniably existing at present and those likely to result from the general adoption of State Licensing Boards by the several States. To this end we must briefly discuss the relations of medicine, as a science and as a profession, to the human race.

It is generally conceded that man, wherever found, wherever studied, is practically the same in his anatomical and physiological peculiarities, or at least that, medically considered, one race of men differ no more from another race than one individual of the same race may differ from another. Theologically man is one, and medically man is one.

From this it follows that the field of practice in which the sacerdotal functions can be exercised is world-wide, and so should be the sphere of the medical practitioner. A physician competent to practice in England should be free to practice in France, in Germany, America, or any place else. The human economy is the same, and the principles which guide the practitioner are the same everywhere. There certainly is no just reason why the competency of a physician to practice his profession should be determined by geographical lines. Dr. Marion Sims was quite as skillful a physician in Paris as he was in New York; and so Dr. Kieth is just as good a surgeon in Edinburgh as he would be in Alabama, and the State Board could not make him any better. If Dr. Gross, or Dr. Mott, or Dr. Flint could reappear and locate in Alabama, Arkansas, Mississippi, North Carolina or Virginia, their own pupils, men who inhaled their first medical breath standing at their knees, would sit in judgment upon their fitness to be numbered among the physicians of their States.

This is manifestly absurd, and the only excuse which can be offered for such a condition of things is a distrust of tests of competency applied in other States or countries, or a disbelief in the honesty of the examiners. While the national vanity of some European countries may lead them to form such a conclusion, there is one, at least, in which one of the pleas urged for the exclusion of foreign physicians was that these latter would obtain the fees from their countrymen, who were there as tourists, which would otherwise be paid to the native practitioners.

A medical diploma is valuable—1st. As an evidence of education. In this particular its value is in proportion to the severity of the published programme of examination and the reputation of the examining board for integrity. 2nd. In proportion to the extent and business value of the territory in which it constitutes a license to practice.

An English diploma would be comparatively valueless if Scotland, and Ireland, and Wales, and the Isle of Man, and each of England's thousand colonies, had an independent examining board. If each of the petty principalities constituting the German Empire had an independent Examining and Licensing Board, then would a German diploma be worthless.

To me it seems that no system of medical educational legislation can be complete that does not keep in view the great principle of *the universality of medicine.*

If every State is to exclude the licentiates of every other State, our national associations of general practitioners, or of specialists, are mere shams—each member practically working to make the world believe that he is better than the rest of his fellow-members. If our medical education is only local, then is an international medical congress a delusion. We cannot meet as equals when each at home lords it over all the others, and merely tolerates an association with those whom under other circumstances he seeks to subject to a professional ostracism. Let us, then, be consistent; let us, instead of wrapping around us an impenetrable mantle of self-conceit, rather endeavor so to frame our actions that, while protecting the community from ignorance and charlatanry, we may raise the grade of medical education to a uniform standard the world over, so that the evidences of a qualification having been once obtained, the holder will be free to travel and practice where he pleases. Nothing short of this would be just to the members of the profession. In populous communities the modern mode is to divide the practice of medicine in specialties. The most expert and celebrated specialist might make but a poor showing in an examination as a general practitioner; and, *per*

*contra*, the general practitioner of many years' practice and great practical experience might easily be shown to be but badly posted upon many of the minutiæ of the specialties. In medicine, as elsewhere, the great man is he who "knows everything of something and something of everything;" while the theory upon which we are now acting is that at all times of his life a man knows everything of everything.

Even with the receptive powers at their best, as it is among our young men, it has been found to be impossible to grasp the whole range of medical education at once, and hence has arisen the system of graded examinations, in which but one or two subjects are taken up at a time. Yet we would require of the old, an aptitude and a receptive and retentive power which has been found to be an impossibility in the young. What has proven to be unattainable with the elastic brain of the youth is made a prerequisite with the hardening brain cells of the adult. Evidently, then, if State Examining Boards are to be the rule, the examinations of the older practitioners who might apply should be confined to those practical subjects which arise in daily practice; but even here the specialist would loom up as an unconquerable difficulty. Possibly in the outset of his career he may have served a long apprenticeship as a general practitioner ; but that may have been many years ago, and in all these years, while practicing as a successful specialist, he may not have attended a case of sickness outside his specialty. It is far from probable that many English physicians of the old school could pass a successful examination on surgery. I refer to physicians of the time when, if it was necessary to bleed a patient, a surgeon was called in to perform the operation. As it is, the specialist does not pretend to expertness outside his specialty. It is evident that the establishment of State Examining Boards will tend certainly to develop a condition of professional sciolism.

The establishment of an independent State Examining Board in each State will ultimately be equivalent to the enactment of a professional passport system—the substitution of a modern professional "jail bounds" for the old and obsolete "debtor's jail bounds"—which, if it is not contrary to the law, is certainly antagonistic to the sentiment of the people of the United States.

Clearly, then, unless medical men are satisfied to endure a life-sentence of professional imprisonment within the limits each of his own State, and if at the same time State Examining Boards are regarded as a necessity, there is but one way out of the dilemma, viz., by the institution of a system of interstate representation upon State Examining Boards, and the establishment of an interstate degree, by agreement between the States; but the true remedy is in the founding of a national degree by the government of the United States; and it is to be hoped that in the near future Congress will find it within its powers to authorize the formation of an Examining Board for the United States. This it could easily do, as such a body could be made almost if not entirely self-supporting, or it could be constructed from physicians already in the service of the government, and would thus entail no additional expense upon the people. Naturally this would fall within the scope of the National Board of Health, and this additional duty, if faithfully, fairly and fearlessly executed, would make this body a national blessing, even if no other result should ever accrue from its labors. Upon the examining board could well be placed representatives of the army, of the navy and of the marine hospital service, and graduates could then be made eligible for a certain number of years to positions in either of these services, first in the order of merit, and in case of equal merit, then in the order of seniority; and it is not impossible that other privileges might be granted which would increase the value of this degree to the holder.

With the composition of the examining board, however, we have really nothing to do. All we desire is the establishment of a degree requiring the best possible medical education, and the institution of a board of examiners, the veracity of whose certificate cannot be called into question.

By suitable representations our representatives in Washington might be induced to give this matter proper shape, and bring it before the Senate and Congress of the United States.

Constitutional lawyers have expressed to me the opinion that this action would come well within the clause of the constitution, giving to Congress the power to "provide for the common defense and the general welfare," and as it would not involve any ultimate outlay, it would not be likely to meet any opposition from the "watch-dogs of the treasury."

To insure a high order of education, it might be well to make provisions: 1st. That the grade of examination or qualification required should never be less than the highest established by any country in the world. 2nd. That a grade of examination once established should never be lowered, or a branch of study once added should never be dropped; but as only very extraordinary minds could pass upon such a variety of subjects at one time, graded examinations might be allowed at reasonable intervals.

By the operation of such a law the grade of American medical education would be constantly advancing, could never be lowered, would soon be the standard for other nations to equal, and what is better than all, the evidence of qualification would be unimpeachable, in this country at least.

The natural outcome of the discoveries of the present century of steamboats, railroads, telegraphs and telephones is the unification of the human race in language, manners and customs, and with the increasing rapidity and facility of travel, the time is rapidly approaching when the convenience of the communities and the interest of humanity will imperatively demand the formation of an International Medical Union between the civilized nations of the world, and the establishment by all the governments interested, of an International Medical Degree, carrying with it the right to practice in all the countries represented in the Union; to this the establishment of an American National Degree is but a preliminary and necessary step. The details of such an institution would simply be a matter of agreement between governments, presenting very much less difficulties than the postal union or extradition treaty, while conferring advantages quite as great.

Of course there would be in each country a system of examining boards, upon which would be representatives of each of the signatory nations, whose powers would necessarily be defined by the treaty.

Not the least of the advantages of the institution of such a degree is that it leaves the educational machinery at present existing in each country absolutely undisturbed; it does not enter into competition with the teachers; it does not abridge the power or privileges or corporate rights of any legally authorized licensing body now in existence, but leaves them all untrammeled in their present sphere of action; calling for a higher and more extended education, it would increase the work for the teachers; in fact, it would enter upon an entirely new and untouched field of operation, and should command the support of every branch of the profession—student, practitioner, teacher and examiner.

The very constitution of such boards of examiners would place them beyond the possibility of having their judgment warped by improper influences, pecuniary, social or political, while the possibility of the examinations assuming a routine form would be guarded against by the frequent changes which would of necessity occur in the *personnel* of the boards, or else the questions presented by the representatives could originate at the seats of government of each of the signatory powers, each propounding a certain number of questions upon each subject.

The furtherance of this object might well occupy a share of the attention of our National and State Medical Associations now disturbed and distracted by valueless questions of ethics, which, when settled, no matter how, will be of no earthly importance, and will not advance the cause of medical science in the least. No more important subject could be brought before the International Medical Congress than the establishment of an International Degree, nor could a greater boon be conferred upon the profession of the world than the institution of such a qualification; in fact, it is the natural outcome of such a congress; it is a legitimate object of discussion in that body, and such a result would be to it an everlasting monument of its wisdom and desire for professional advancement of which it might well be proud, and for which future generations would "lift up their hands and call it blessed;" or again, the Society of the Red Cross, which, from the very nature of the work in which it is engaged, and from the wide sphere of its operations, would be interested in this matter, might be induced to turn its attention and use its influence in this direction, to the furtherance of this object.

The closer and more intimate the relations between nations, the less likelihood will there be of war; the more numerous the threads which hold communities together, the less will be the probability of rupture. The establishment of an International Medical Degree will be one more link in the chain of civilization which binds nationalities, increasing the intimacy of their relations to each other, and putting farther and farther away the probabilities of war, or if war does come, tending to alleviate many of its horrors. It would be a freemasonry of science. Represented in this light, the effort to establish an International Medical Degree would doubtless be actively and warmly seconded by the members of the Peace Congress. Viewing the matter in its various aspects, it seems not improbable that the effort to found such a degree would find a cordial and numerous support throughout the world.

To the individual physician the possession of such a degree would be of inestimable value, and it would unquestionably be ardently sought after. It would confer upon the holder a freedom of action which he could acquire in no other way, and would indelibly stamp his educational qualification as equal to any in the world; it would give to the youngest practitioner the highest standing possible to attain, and make him instantly, by force of his own genius, a world-wide authority in medical matters.

To communities such a degree would be scarcely less valuable than to the graduate. It would enable invalids who so desired to bring to their assistance medical men from any quarter; and in times of epidemic or war, medical aid could be summoned, if needed, which, under the State law, or national system, would be illegal, although doubtless under such circumstances it would be tolerated.

With the institution of an International Medical Degree we would see a mighty scientific brotherhood start into existence in which truly the principles of liberty, equality and fraternity could be legitimately carried out; a great republic of science would be born into the world, covering a territory co-extensive with civilization. A power for good would leap forth, before which the mightiest of kingdoms or governments would dwindle into insignificance.

Cannot the Medical Association of Georgia initiate a movement which may ultimate in so great a result? Can we not secure for our grand old State the credit and the immortal glory of sowing the seed which will bring forth fruit of untold blessings to humanity? Gentlemen, members of the Medical Association of Georgia, it is given to you to say whether this shall or shall not be.

Your attention has been directed in this channel from a desire to do away with these purely local ideas which confine our educational schemes to the narrow limits of a State or a country, from a desire to broaden our philanthropy until it embraces the whole profession of the world, from a desire to elevate our estimate of medicine until we can look upon the profession as a unit, whether in Russia or in the United States, whether in Canada or Australia.

It is not to be denied that this proposition savors of Utopia, but it is no more ideal than the postal union, no more impracticable than extradition or any other international treaty.

May we, before we return our trust to the Great Giver, be able to look upon one medical profession wide-spread as civilization, whose members will not be limited in the exercise of their functions by any geographical or governmental boundaries. May we live to see the day when suffering humanity can summon to its assistance skilled physicians of any nationality who will be free to exercise their profession untrammeled by local laws, begotten of a narrow, contracted, erroneous interpretation of the duties and requirements of the medical profession. When that day arrives a new era will dawn upon medicine; then will the profession assume an importance now not dreamed of; then will it become a power of enormous social influence; its now heterogeneous elements will then be aglutinated into one harmonious whole, working together for their common good, and for the welfare and advancement of civilization and of humanity.

Gentlemen, members of the Medical Association of Georgia, the duty assigned to me by your constitution has been performed. The problem which was suggested at the beginning of this address is solved, and in an International Medical Degree we have a qualification far removed from the influences of illiteracy and ignorance, and unattainable by the charlatan and the quack. We have the means of insuring to communities physicians possessing the highest attainable degree of medical knowledge and expertness, and we have the power of giving to persons having the requisite qualifications such evidences of the fact as shall pass unquestioned and unquestionable over the world.

